# Localized Pulmonary Amyloidosis Associated With Sjögren’s Syndrome, Coexisting Lymphoid Interstitial Pneumonia, and a Severe Double Aortic Lesion: A Case Report and Literature Review

**DOI:** 10.7759/cureus.103714

**Published:** 2026-02-16

**Authors:** Felipe-de-Jesus Horta-Saucedo, Dulce-Iliana Navarro-Vergara, Maria-Berenice Torres-Rojas, Alonso Gonzalez-Ares, Ana Alfaro-Cruz, Alejandro Altamirano-Jiménez, Carlos-Joaquín Pech-Lugo, Daniel Ruiz-Domínguez, Raul Reynoso-Ruelas, Ernesto Roldan-Valadez

**Affiliations:** 1 Pulmonary Circulation Clinic, Hospital General de México 'Dr. Eduardo Liceaga', Mexico, MEX; 2 Cardiorespiratory Emergencies, Hospital General de México 'Dr. Eduardo Liceaga', Mexico, MEX; 3 Pathological Anatomy Department, Hospital General de México 'Dr. Eduardo Liceaga', Mexico, MEX; 4 Research, Instituto Nacional de Rehabilitacion "Luis Guillermo Ibarra Ibarra" (INR), Mexico, MEX

**Keywords:** amyloidosis al, aortic valve stenosis, congo red stain, high-resolution computed tomography (hrct), lymphoid interstitial pneumonia, pulmonary hypertension, pulmonary nodules, sjögren’s syndrome

## Abstract

Amyloidosis is a protein-folding disease that results in the deposition of misfolded proteins into various tissues of the body, resulting in dysfunction of the affected organ. It is caused by a heterogeneous group of disorders caused by extracellular deposition of misfolded proteins as insoluble fibrils. Involvement of the lungs can present as nodular, tracheobronchial, or diffuse interstitial, and can be mistaken for cancer or infection on imaging. The combination of Sjögren's syndrome, chronic activation of B cells, and the risk of developing a lymphoproliferative disorder can lead to the rare deposition of amyloid. In cystic lung disease, the amyloid deposition can be misattributed to lymphoid interstitial pneumonia (LIP).

An evaluation for progressive dyspnea and chest pain was done in a 68-year-old female patient diagnosed with Sjögren's syndrome and severe degenerative aortic valve disease (severe double aortic lesion). The patient’s high-resolution computed tomography scan demonstrated diffuse pulmonary cysts of the thin-walled variety that are consistent with a pattern of LIP, with several solid nodules layered on top of each other exhibiting increased attenuation, as well as some that are calcified on the mediastinal windows. An October 2023 core lung biopsy revealed structures resembling bony spicules with dystrophic calcification and an adjacent amorphous area that lacked cells; malignancy was absent. Microbiological studies were negative for bacteria, fungi, and mycobacteria, and there were no malignant cells in the cytology. The patient's autoimmune testing revealed rheumatoid factor at 20.2 IU/mL, as well as anti-Ro (SSA) 125 and anti-La (SSB) 79.9. Sjögren's syndrome was confirmed by a salivary gland biopsy.

Re-reviewing archived biopsy material in 2025 showed that the unexplained nodules still had atypical radiographic characteristics, with the Belizean red stain showing polarized light birefringence, confirming the presence of pulmonary amyloid. The functional assessment showed a 6-minute walk distance of 392 m with an oxygen saturation nadir of 85% at minute 4. Right heart catheterization with fluid challenge (August 2025) showed a mean pulmonary artery pressure of 24 mmHg, pulmonary artery wedge pressure rising from 12 to 18 mmHg, hypothesized cardiac output of 5.1 L/min, cardiac index 3.6 L/min/m², and pulmonary vascular resistance of 2.3 Wood units, consistent with mild pulmonary hypertension and a post-capillary contribution with severe aortic valve disease. The patient continues to be managed by a multidisciplinary team, with no systemic amyloidosis documented to date.

When pulmonary nodules co-exist with Sjögren’s syndrome, pulmonary amyloidosis, although rare, should be considered, especially when the syndrome co-exists with cystic changes that suggest LIP. Persistent atypical changes in radiology and biopsy should trigger a more thorough examination of stale biopsies. Secondary severe valve disease, augmenting pulmonary hypertension, should be an impetus for ongoing multidisciplinary care and a thorough ongoing assessment.

## Introduction

Systemic amyloidosis includes a diverse collection of diseases that result in the extracellular misfolding of proteins in insoluble fibrils that are deposited in the tissues, which ultimately leads to progressive failure of organs [1]. Foundational population-based studies are the first to establish early benchmarks in epidemiologic studies of amyloid light-chain (AL) amyloidosis in certain cohorts [2]. Recent studies with registries and national datasets show that systemic amyloidosis is rare; however, it is more prevalent in Western countries [3-5]. The prognosis of AL amyloidosis is heavily impacted by the presence and extent of involvement of particular organs, as deposition of the proteins in the heart is one of the most significant causes of the disease’s morbidity and mortality [6].

Amyloidoses can be categorized by their precursor proteins, whether they are localized or systemic, and their underlying causes; these classifications also provide directions for the diagnostic procedures and predicted patterns of organ involvement. The most common types are AL (primary), amyloid A (AA; secondary), wild-type transthyretin (ATTR) amyloidosis, hereditary transthyretin (ATTR) amyloidosis, and localized AL. The affected organs can be the kidneys, heart, peripheral nerves, liver, spleen, gastrointestinal tract, skin, and lungs, and systemic disease is often suspected based on the involvement of multiple organs [7].

Confirmation of the diagnosis must come from histopathological studies. Apple-green birefringence under polarized light with coining red staining is still a reference standard; stains like crystal violet or thioflavin T may have some merit as supportive stains [7-9]. As management considerably varies by type of amyloidosis, subtyping is of clinical relevance; evaluation for a concomitant monoclonal gammopathy typically entails serum immunofixation and electrophoresis [10]. Imaging has a role in disease mapping and long-term follow-up; in cases of thoracic involvement, CT is especially valuable in defining the morphology of the lesions and monitoring interval changes over time [11].

Pulmonary amyloidosis may present as solitary or multiple nodules or masses, tracheobronchial disease, or diffuse interstitial patterns. From a diagnostic perspective, nodular pulmonary amyloidosis presents unique challenges as CT findings may mimic malignancy or infection, and lesions may be hypermetabolically active on positron emission tomography (PET), resulting in oncologic workup unless there is early consideration of amyloid. Prior studies have noted a range of presentations from single lesions to multiple nodules [12,13]. This difference is important to try to determine the course of localized pulmonary amyloidosis, which may remain stable for a period of time; however, the course of systemic disease is more problematic and warrants evaluation for systemic diseases or other organ involvement to determine a more precise management plan [7,9]. In cases where the clinical course and imaging studies do not seem to align with the initial reading of the pathology, the systematic review of the tissues using amyloid-specific stains and typing, when applicable, may prove to be decisive in avoiding or correcting the diagnosis when a delay is present [14].

Association between Sjögren’s syndrome and amyloidosis

Sjögren’s syndrome is a chronic autoimmune disease that has been linked to B-cell hyperactivity and dysregulation of the immune system. This scenario can lead to a condition called monoclonal gammopathy, along with lymphoproliferative disorders, such as mucosa-associated lymphoid tissue (MALT) lymphoma, and provides a biologic substrate for the production of immunoglobulin light chains, and, in some cases, AL-type amyloid [15]. Amyloid deposits can be localized to a single organ and can be systemic, with the possible involvement of solid organs such as the salivary glands, lungs, and kidneys. Patients may present with glandular swelling, pulmonary nodules, and even nephrotic syndrome [16]. This is highly relevant to clinicians, as in the case of Sjögren’s syndrome, if patients have pulmonary nodules that are persistent or atypical, the possibility of amyloidosis must be considered, and for prognosis and management, a Congo red-stained biopsy with amyloid typing is critical [14].

## Case presentation

A 68-year-old married woman (with primary education) was being examined for chronic pulmonary disease with increasing cardiopulmonary symptoms. She had a history of 10 years of childhood exposure to a poultry farm and had a proven allergy to quinolones. Her medical history was significant for severe degenerative aortic valve disease, described as a severe double aortic lesion, and longitudinal follow-up in pulmonology for lymphoid interstitial pneumonia (LIP). Major events in her clinical pathway are presented in Table 1.

**Table 1:** Event chronology in clinical practice, diagnostic studies, and hemodynamics in pulmonary amyloidosis with associated Sjögren’s syndrome Chronology of significant clinical events, relevant imaging, specific biopsies, follow-up evaluations, confirmed autoimmune disorder, consolidated cardiac evaluation, and direct measurements of the right heart (via catheterization). Relevant high-resolution CT (HRCT) findings are depicted in Figure 1, and the Congo red stain confirming amyloid deposition in Figure 2.

Timepoint	Clinical context	Key investigations	Main findings	Interpretation/action
Childhood (10 years)	Environmental exposure	History	Childhood exposure to poultry farming for approximately 10 years	Exposure history documented
Baseline (prior to 2022)	Chronic cardiopulmonary comorbidity	Clinical follow-up	Severe degenerative aortic valve disease (“severe double aortic lesion”) and longitudinal pulmonary follow-up for lymphoid interstitial pneumonia (LIP)	Ongoing multidisciplinary care
2022	Symptom progression	High-resolution computed tomography (HRCT)	Multiple pulmonary nodules with diffuse thin-walled cysts reported as compatible with an LIP pattern (Figures 1A-1C)	Imaging phenotype initially framed within the Sjögren-related interstitial lung disease spectrum
Oct 2023	Persistent radiologic abnormalities	Tru-cut (core needle) lung biopsy; microbiology and cytology	No malignancy identified; ancillary studies negative for bacteria, fungi, and mycobacteria; cytology negative for malignant cells	No neoplasia or infection was demonstrated on initial sampling
2023 (work-up period)	Autoimmune suspicion	Serology and cardiac biomarker; salivary gland biopsy	Rheumatoid factor 20.2 IU/mL; anti-Ro (SSA) 125; anti-La (SSB) 79.9; pro–B-type natriuretic peptide (proBNP) 54 ng/mL; salivary gland biopsy confirmed Sjögren’s syndrome	Autoimmune diagnosis established; cardiopulmonary surveillance continued
Nov 2024	Valvular disease reassessment	Transthoracic echocardiography	Findings consistent with severe aortic valve disease; no echocardiographic pattern typical of infiltrative cardiomyopathy on available assessment	Cardiac findings were attributed primarily to valvular pathology in available assessment
2025	Diagnostic reconsideration due to persistent nodular disease	Re-evaluation of archived lung biopsy material with special stains	Congo red staining demonstrated birefringence under polarized light in amorphous material, confirming pulmonary amyloid deposition (Figure 2C)	Diagnosis revised to pulmonary amyloidosis; clinico-radiologic–pathologic correlation updated
Aug 2025	Suspected combined pulmonary hypertension	Right heart catheterization with fluid challenge	Mean pulmonary artery pressure 24 mmHg; pulmonary artery wedge pressure 12 mmHg rising to 18 mmHg after fluid challenge (reported as positive); cardiac output 5.1 L/min; cardiac index 3.6 L/min/m²; pulmonary vascular resistance 2.3 Wood units	Hemodynamics consistent with mild pulmonary hypertension with a postcapillary contribution in the context of severe left-sided valvular disease
Current	Longitudinal management	Multidisciplinary follow-up	Ongoing cardiology and rheumatology follow-up	Working clinical synthesis: pulmonary amyloidosis in the setting of Sjögren’s syndrome, coexisting LIP, severe double aortic lesion, and mild pulmonary hypertension physiology

**Figure 1:**
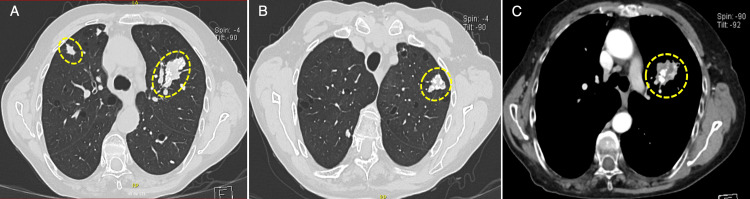
High-resolution computed tomography of the chest demonstrating pulmonary cysts and nodules (A) Axial non-contrast lung-window image shows numerous thin-walled pulmonary cysts (approximately 1-30 mm) with basal predominance (yellow dashed circles). (B) Axial contrast-enhanced mediastinal-window image again highlights the diffuse cystic changes (yellow dashed circles). (C) Axial contrast-enhanced mediastinal-window image shows two solid, heterogeneous pulmonary nodules with internal calcified foci and heterogeneous enhancement in the left apical and apicoposterior segments (yellow dashed circle).

**Figure 2:**
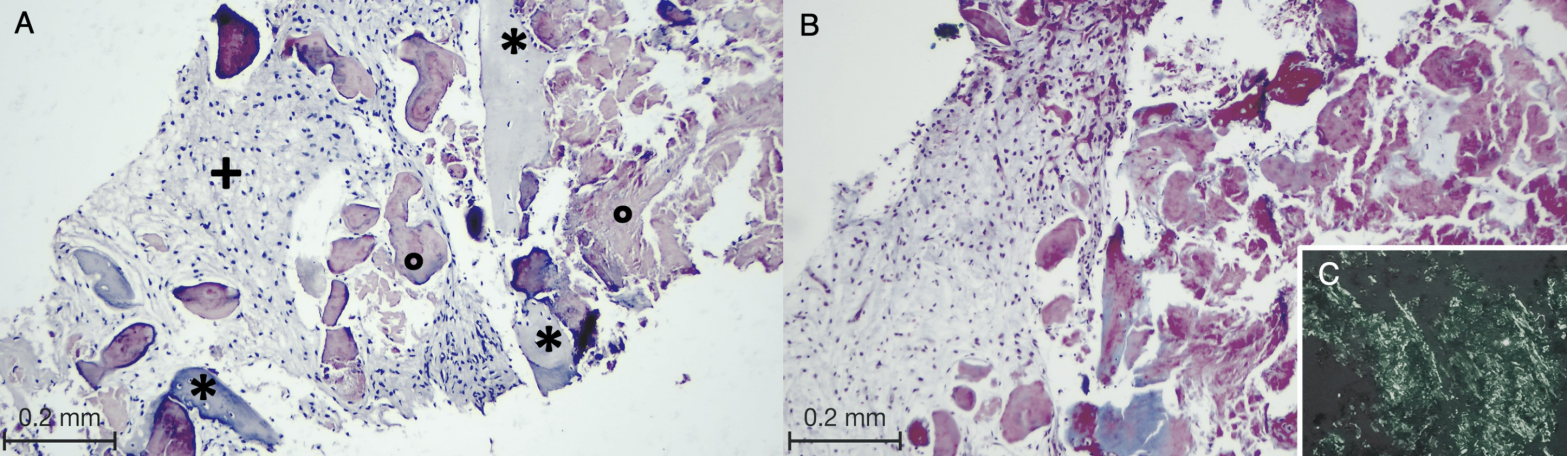
Lung biopsy histopathology showing amyloid deposition (A, B) At low magnification, Masson’s trichrome stain reveals bony spicules (asterisks) with dystrophic calcification and surrounding acellular, amorphous material (open circles). The adjacent lung parenchyma is replaced with fibrosis (plus signs). (C; lower inset) Congo red stain shows the characteristic birefringence of the amorphous deposits consistent with amyloid under polarized light.

In 2022, she started having increasing difficulty breathing and also had chest pain. A high-resolution chest CT (HRCT) showed the presence of multiple pulmonary nodules with diffuse cysts, interpreted to be concordant with the LIP pattern; multiple nodules were noticed to have increased attenuation with calcified components on the mediastinal windows (Figures 1A-1C; Table 1).

A Tru-Cut (core needle) lung biopsy was done in October 2023 due to the persistence of the nodular component. The initial pathology report was still negative for malignancy, and the additional tests were negative for bacteria, fungi, and mycobacteria, and the cytology report was negative for malignant cells. In the course of the additional autoimmune assessment, the serum rheumatic factor was 20.2 IU/mL, anti-Ro (SSA) and anti-La (SSB) were 125 and 79.9, respectively, and the pro B-type natriuretic peptide (proBNP) was 54 ng/mL. The biopsy of the salivary glands was indicative of Sjögren's syndrome (Table 1).

In November 2024, the transthoracic echocardiography showed findings of severe aortic valve disease, and there were no echocardiographic signs of infiltrative cardiomyopathy on the available evaluation. In 2025, given the persistence of pulmonary nodules and the overall clinicoradiologic course, the archived lung biopsy specimens were restudied with special staining. The Congo red histochemistry showed the typical bifringence under polarized light of acellular amorphous material, confirming pulmonary deposition of amyloid. The tissue fragments showed the presence of bony spicules and dystrophic calcification (Figures 2A-2C; Table 1).

The 6-minute walk test for functional assessment indicated a distance of 392 m, with a recorded oxygen saturation of 85% at minute 4. Standard pulmonary function tests, including spirometry, lung volume, and diffusing capacity for carbon monoxide (DLCO), were unacceptable, so oscillometry was done, revealing mild proximal airway obstruction.

Given the suspicion of combined pulmonary hypertension, our group performed cardiac catheterization in August 2025 using the standard methodology described in our previous work [17]. A mean pulmonary artery pressure of 24 mmHg and a pulmonary artery wedge pressure of 12 mmHg were recorded, with a positive fluid challenge increasing the wedge pressure to 18 mmHg. Cardiac output was 5.1 L/min, the cardiac index was 3.6 L/min/m², and pulmonary vascular resistance was 2.3 Wood units. The patient continues multidisciplinary follow-up with cardiology and rheumatology, and the summed diagnoses were pulmonary amyloidosis in the context of Sjögren's syndrome, coexistent LIP, double aortic lesion, and combined pulmonary hypertension (Table 1; Figures 1-2).

## Discussion

The rare condition pulmonary amyloidosis often results in delayed diagnosis, contributing to its diagnostic challenges as pulmonary amyloidosis can act as both a radiological and clinical mimic [9,12,13,18]. Pulmonary amyloidosis can act as a clinical and radiological mimic, which explains why HRCT (high-resolution computed tomography) scans, where pulmonary amyloidosis presents with cysts and nodules, are often mistaken for other conditions. For this case, HRCT showed a pattern of cysts and lacked nodules, which posed a differential diagnosis of Sjögren's syndrome and LIP [15,19]. The most frequent inconsistencies of this diagnosis are solid nodules, which can make mediastinal HRCT windows appear hyperdense, with calcified foci (gray scale imaging) and give rise to non-cancer/infection previous biopsy interpretations (Table 1). In this case, re-visiting and correlating the clinical-radiological-pathological context with archived re-evaluated tissues was the best way to arrive at a more certain diagnosis.

The effectiveness of a phenotype-based framework becomes evident with pulmonary amyloidosis's heterogeneity, as it can present with localized nodular disease, disease with bronchial involvement, cystic and nodular phenotypes in combination, and the diffuse deposition of amyloid in the alveolar septae [18]. Of particular relevance for daily practice is localized nodular pulmonary amyloidosis because CT findings can mimic primary lung malignancy, metastatic disease, or granulomatous infection, and it can show increased uptake on positron emission tomography, which may lead to an oncological evaluation unless amyloid is considered early. In published series, nodules may be solitary or multifocal, variably calcified, and indolent or slowly progressive, so longitudinal imaging behavior is an important contextual clue [12,13].

The association of Sjögren’s syndrome and a LIP-compatible cystic phenotype may result in a diagnostic trap: once autoimmune interstitial lung disease is assumed, superimposed processes may be overlooked [15,16]. This is important because Sjögren’s syndrome has persistent B-cell activation and an increased risk of monoclonal gammopathy and lymphoproliferative disorders, including MALT lymphoma, which provides a biologic substrate for light-chain and, in select cases, AL-type light-chain amyloidosis [15,16]. Thus, pulmonary nodules with features that are atypical for an inflammatory process, such as conspicuous hyperdensity, calcification, persistence, or progressive enlargement, should keep amyloidosis in the differential diagnosis, along with malignancy and infection, even in the presence of a cystic background consistent with LIP (Figure 1; Table 1) [12-14]. The imaging heterogeneity of biopsy-proven pulmonary amyloidosis lends support to a low threshold for obtaining tissue diagnosis when the behavior of a lesion is inconsistent with the presumed diagnosis. In this situation, the LIP-like cystic pattern and calcified nodules demonstrate how anchoring to the autoimmune diagnosis may postpone recognition of a second process (Figure 1; Table 1) [19].

When amyloid is not suspected initially, pathology will provide the key clarification. Core biopsies can show calcifications or amorphous material, which can be misclassified, especially in instances of limited sampling with malignancy being the primary clinical concern. The review of previously unexamined biopsy material showed amyloid deposits via Congo red staining, which showed bifringement when viewed with polarized light (Figure 2), revising the longitudinal clinical course of the patient (Table 1) [7-9,14,20]. This is an important step, as it could minimize diagnostic drift, possibly eliminating the need for more invasive procedures when clinical imaging does not correlate with the initial pathology [14].

When there are multiple cardiopulmonary mechanisms involved, hemodynamic characterization is critical. Right heart catheterization in this patient recorded a mean pulmonary artery pressure of 24 mmHg, which complies with the most recent hemodynamic definition of pulmonary hypertension [21-23], with pulmonary artery wedge pressure increasing from 12 mmHg to 18 mmHg post fluid challenge and pulmonary vascular resistance being only mildly elevated (2.3 Wood units) (Table 1). This combines the post-capillary effects of mild pulmonary hypertension with severe aortic valve disease, and rather than isolated precapillary pulmonary vascular disease [9], suggests post-capillary predominance. In Sjögren’s syndrome, pulmonary hypertension has been recorded for several reasons, which also include lung parenchyma, left-sided heart disease, and pulmonary vascular disease, and so when it comes to invasive hemodynamics, for the most part, the goal is to prove the theory correct [15].

In light of the above, this case illustrates three important points. First, for Sjögren’s syndrome with cystic lung disease consistent with LIP, persistent pulmonary nodules, especially if hyperdense or calcified, should include the consideration of pulmonary amyloidosis in addition to malignancy or infection, and be the basis for further reassessment if the clinical course defies the presumed diagnosis (Figure 1; Table 1) [12-15]. Second, when imaging change remains inconsistent with a given biopsy, a systematic approach to re-archiving the tissues with Congo red stain may be a defining factor (Figure 2; Table 1) [7-9,14,20]. Third, in the context of autoimmune disease, when left-sided valvular disease is concomitantly suspected, pulmonary hypertension is presumed, and thus, right heart catheterization will elucidate the mechanism and provide a basis for rational multidisciplinary management that extends beyond single-tract management [9].

This report is limited in scope to a single patient, and the Congo red amyloid subtype characterization, or the absence of it, limits the scope of questions on potential etiology and systemic risk. Regardless, this case exemplifies how overlapping imaging patterns associated with autoimmunity may obscure a second process, and how focused clinico-radiologic-pathologic reconciliation, with the right stain and the appropriate time period, may significantly alter the understanding of the diagnosis and the management.

## Conclusions

In patients with Sjögren’s syndrome and certain cystic lung patterns consistent with lymphoid interstitial pneumonia, solid pulmonary nodules, combined with increased solid attenuation and calcified foci on high-resolution CT scans, lead to multiple diagnostic changes and eventually to a diagnosis of pulmonary amyloid deposition after a review of archived lung biopsies with Congo red staining. This case demonstrates that overlapping autoimmune-associated imaging phenotypes can obscure a superimposed process, and emphasizes the importance of strict clinico-radiologic-pathologic correlation when the longitudinal course is incongruent with the working diagnosis. 

In patients with an autoimmune disease, pulmonary and aortic valve disease, postcapillary mild pulmonary hypertension, and concomitant moderate pulmonary hypertension, the role of right heart catheterization is reinforced to explain the mechanisms of the disease. In patients with autoimmune disease and complex cardiopulmonary comorbidity, the integration of hemodynamics with imaging and histopathology allows for rational multidisciplinary management with longitudinal follow-up and amyloid subtyping.
